# Adipose-Derived Mesenchymal Stem Cell Facilitate Hematopoietic Stem Cell Proliferation via the Jagged-1/Notch-1/Hes Signaling Pathway

**DOI:** 10.1155/2023/1068405

**Published:** 2023-11-09

**Authors:** Hongbo Wang, Xiaojuan Bi, Rongyao Zhang, Hailong Yuan, Jianli Xu, Kaile Zhang, Songqing Qi, Xue Zhang, Ming Jiang

**Affiliations:** ^1^Hematology Center, The First Affiliated Hospital of Xinjiang Medical University (Xinjiang Uygur Autonomous Region Institute of Hematology), Urumqi 830054, China; ^2^Stem Cell Research Center, The First Affiliated Hospital of Xinjiang Medical University, Urumqi 830054, China; ^3^The State Key Laboratory of Pathogenesis and Prevention of Central Asian High Incidence Diseases, Institute of Clinical Medicine, The First Affiliated Hospital of Xinjiang Medical University, Urumqi 830054, China

## Abstract

**Background:**

Poor graft function (PGF) is a life-threatening complication following hematopoietic stem cell transplantation (HSCT). Current therapies, such as CD34^+^ cell infusion, have shown limited effectiveness. Conversely, mesenchymal stem cells (MSCs) show potential in addressing PGF. Adipose-derived mesenchymal stem cells (ADSCs) effectively support long-term hematopoietic stem cell proliferation. Therefore, this study aimed to investigate the mechanisms underlying the long-term hematopoietic support provided by ADSCs.

**Methods:**

ADSCs were isolated from mice and subsequently identified. *In vitro* experiments involved coculturing ADSCs as feeders with Lin-Sca-1^+^c-kit^+^ (LSK) cells from mice for 2 and 5 weeks. The number of LSK cells was quantified after coculture. Scanning electron microscopy was utilized to observe the interaction between ADSCs and LSK cells. Hes-1 expression was assessed using western blot and real-time quantitative PCR. An *γ*-secretase inhibitor (GSI) was used to confirm the involvement of the Jagged-1/Notch-1/Hes-1 pathway in LSK cell expansion. Additionally, Jagged-1 was knocked down in ADSCs to demonstrate its significance in ADSC-mediated hematopoietic support. *In vivo* experiments were conducted to study the hematopoietic support provided by ADSCs through the infusion of LSK, LSK + fibroblasts, and LSK + ADSCs, respectively. Mouse survival, platelet count, leukocyte count, and hemoglobin levels were monitored.

**Results:**

ADSCs showed high-Jagged-1 expression and promoted LSK cell proliferation. There was a direct interaction between ADSCs and LSK cells. After coculture, Hes-1 expression increased in LSK cells. Moreover, GSI-reduced LSK cell proliferation and Hes-1 expression. Knockdown of Jagged-1 attenuated ADSCs-mediated promotion of LSK cell proliferation. Furthermore, ADSCs facilitated hematopoietic recovery and promoted the survival of NOD/SCID mice.

**Conclusion:**

The hematopoietic support provided by ADSCs both *in vivo* and *in vitro* may be mediated, at least in part, through the Jagged-1/Notch-1 signaling pathway. These findings provide valuable insights into the mechanisms underlying ADSCs-mediated hematopoietic support and may have implications for improving the treatment of PGF following HSCT.

## 1. Introduction

Allogeneic hematopoietic stem cell transplantation (HSCT) is an effective means to treat hematological diseases. However, poor graft function (PGF), with an incidence of 10%–20%, is a common complication after transplantation, which is closely related to the damage of the bone marrow microenvironment after radiotherapy and chemotherapy [1, 2].

The bone marrow niche, which is composed of bone marrow endothelial stromal cells, osteoblasts, bone marrow mesenchymal stem cells (BMSCs) etc., plays a vital role in the homing, proliferation, and differentiation of hematopoietic stem cells [3]. Of note, BMSCs can provide certain supporting effects on hematopoiesis [4]. However, in the clinical practice, BMSCs may weaken the anti-leukemia effect of grafts and increase the risk of recurrence [5]. Adipose-derived mesenchymal stem cells (ADSCs) were first identified by Zuk et al. [6] in 2002. ADSCs and BMSCs have similar biological characteristics [7] but have unique mechanisms in immune regulation and differentiation. ADSCs have less adverse impact on the proliferation and function of natural killer (NK) cells, and thus more GVL effects may be retained [8]. Meanwhile, ADSCs can secrete more IL-33, which can bind to ST-2^+^ Treg cells to promote their proliferation and suppress excessive immune responses [7]. ADSCs have been mainly applied in orthopedics, but little attention is paid to their role in the hematopoietic system [9, 10]. In our previous study, we found that ADSCs promoted the proliferation of rat hematopoietic stem cells *in vivo* and *in vitro* for a long time and compared with BMSCs, ADSCs have a stronger ability to promote hematopoiesis [10]. However, the mechanisms underlying the role of ADSCs in hematopoiesis remain largely unknown.

It has been confirmed that mesenchymal stem cells (MSCs) together with other important components such as osteoblasts, reticular matrix, and vascular/sinus endothelial cells in bone marrow can maintain the proliferation and differentiation of hematopoietic stem/progenitor cells by providing growth factors and secreting matrix proteins [11] and via cell–cell contact [12]. The Jagged/Notch signal pathway is a highly conservative signal pathway that transmits signals through cell–cell contact, and Jagged-1 plays an important role in maintaining self-renewal and differentiation of hematopoietic stem cells [13–15]. However, whether the Jagged/Notch signal pathway is involved in the role of ADSCs in hematopoiesis is unclear.

Herein, we investigated the role of ADSCs in hematopoiesis. The mechanism involving the Jagged/Notch signal pathway was analyzed and discussed. Our findings may provide experimental evidence for the application of ADSCs in hematopoiesis and for improving PGF after HSCT.

## 2. Materials and Methods

### 2.1. Animals

NOD/SCID mice (*n* = 30; age 8 weeks; body weight 22–28 g; female) (Beijing Vital River Laboratory Animal Technology Co., Ltd., China) and C57/BL mice (*n* = 20; age 8 weeks; body weight 25–28 g; male) (the Animal Center of Xinjiang Medical University) were used in this study. All animals were kept in standard conditions. All animal experimental procedures were approved by the Ethics Committee of the First Affiliated Hospital of Xinjiang Medical University.

### 2.2. Isolation and Identification of ADSCs

ADSCs were isolated from the inguinal adipose tissues of male C57/BL mice (*n* = 3), according to the previous description [10]. In brief, the inguinal adipose tissues were obtained under aseptic conditions. After removing the blood vessels, lymph nodes, and fascia, the adipose tissues were cut into small pieces (1 mm^3^) and digested with 0.1% type I collagenase (cat.# LS004200; Worthington Biochemical Corp., NJ, USA) at 37°C for 30 min. Then, the samples were centrifuged at 2,000 r/min for 10 min, and the cells in the precipitate were collected, resuspended, and cultured in the low-glucose Dulbecco's modified Eagle's medium (DMEM) (cat.# SH30021.FS; Hyclone. UT, USA) medium containing 10% fetal bovine serum (FBS) (cat.# 10099-141C; Gibco; Thermo Fisher Scientific, Inc. MA, USA), 100 U/mL penicillin, and 100 mg/mL streptomycin (cat.# SV30010; Hyclone, UT, USA). Cells were plated at a density of 4× 10^4^ cells/cm^2^ in T25 cell culture bottles and kept at a temperature of 37°C, 5% CO_2_, and saturated humidity. After culturing for 48 hr, the culture medium was replaced and the cells were passed at a cell confluence of 80%–90%. The morphology of P3 cells was observed.

To evaluate the cell differentiation ability of ADSCs, P3 cells were, respectively, cultured in the osteogenic medium and adipogenic medium [16]. The adipogenic medium was composed of low-glucose DMEM supplemented with 10% FBS, 0.1 *µ*mol/L dexamethasone (cat.# HY-14648; MedChemExpress, NJ, USA), 200 *µ*mol/L indometacin (cat.# HY-14397; MedChemExpress, NJ, USA), 10 *μ*mol/L insulin (cat.# HY-P0035; MedChemExpress, NJ USA), and 0.5 mmol/L 3-isobutyl-1-methylxanthine (cat.# HY-12318; MedChemExpress, NJ, USA). The osteogenic medium consisted of low-glucose DMEM, 10% FBS, 0.1 *µ*mol/L dexamethasone, 50 *µ*mol/L ascorbic acid (cat.# HY-103701 A; MedChemExpress, NJ, USA), and 10 mmol/L sodium *β*-glycerophosphate (cat.# HY-126304; MedChemExpress, NJ, USA). After culturing at 37°C, with 5% CO_2_ and saturated humidity for 2 weeks, ADSCs were fixed with 4% paraformaldehyde and washed twice with phosphate buffers (PBS) (cat.#SH30256.FS, UT, USA). Then, they were stained with 1% Alizarin Red (cat. #A5533, Sigma–Aldrich, Darmstadt, Germany) at a pH of 4.2 over a period of 3 min and 0.5% Oil red O (cat. #O1391, Sigma–Aldrich, Darmstadt, Germany) for 10 min, respectively, to determine their multidirectional differentiation ability. Meanwhile, P3 ADSCs were trypsinized using 0.25% trypsin and prepared as a single-cell suspension. The suspension was centrifuged at 1,000 *g* for 5 min, and the supernatant was discarded. The cells were then washed twice with PBS. ADSCs (1 × 10^6^) were incubated with monoclonal antibodies at 4°C in the dark for 30 min. The antibodies included: CD34-PE (cat. # 119307; BioLegend, SanDiego, CA, USA), CD105-PE (cat. # 120407; BioLegend, SanDiego, CA, USA), CD45-FITC (cat. # 157607; BioLegend, SanDiego, CA, USA), and CD29PE (cat. # 102207; BioLegend, SanDiego, CA, USA). Finally, the cells were detected on the CytoFLEXV2B4R0 flow cytometer (C02944; Beckman Coulter, Inc. Indianapolis, USA), and the results were analyzed with Kaluza software (Beckman Coulter, Inc. Indianapolis, IN, USA).

To assess the proliferation of ADSCs, P3 cells were plated into a 96-well plate (500 cells per well) and cultured in a 5% CO_2_ incubator at 37°C. On Days 1, 2, 3, 4, 5, 6, 7, and 8 of the culture, cells from three wells were collected and counted. Cell growth curves were plotted.

### 2.3. Isolation and Identification of Lin^−^Sca-1^+^c-Kit^+^ (LSK)

C57 mice were anesthetized with CO_2_ and euthanized to retrieve bilateral femurs. The femurs were then incubated in a PBS solution containing penicillin (100 U/mL) and streptomycin (0.1 mg/mL) for 20 min. A buffer solution containing PBS (pH 7.2), 0.5% BSA (bovine serum albumin), and 2 mM EDTA (Ethylene Diamine Tetraacetic Acid) was prepared by diluting MACS BSA Stock Solution (cat.# 130-091-376 Miltenyi Biotec, North Rhine-Westphalia, Germany) at a ratio of 1 : 20 with auto MACS™ Rinsing Solution (cat.# 130-091-222, Miltenyi Biotec, North Rhine-Westphalia, Germany) and kept at 4–8°C. The cell suspension was obtained by repeatedly flushing the bone marrow cavities using a 26 G needle. Mouse lymphocyte separation solution (cat.#P8620; Solarbio, Beijing, China) was used for cell isolation, followed by density gradient centrifugation at 2,000 r/min and 20°C for 30 min using a centrifuge (5810R, Eppendorf, Hamburg, Germany). Mononuclear cells were collected, and a resuspension of 1 × 10^7^ mononuclear cells was prepared. Additionally, 10 *μ*L lineage antibody [17] (cat.#130-090-858; Miltenyi Biotec, North Rhine-Westphalia, Germany) was added to the mononuclear cells, followed by incubation at 4°C for 10 min. Subsequently, 30 *μ*L buffer and 20 *μ*L magnetic beads were added, thoroughly mixed, and incubated on ice for 15 min. The cells were washed, followed by centrifugation at 300 *g* for 10 min. The supernatant was then discarded, and the cells were resuspended in 500 *μ*L buffer per 1 × 10^8^ cells. Lin^−^ cells were obtained by passing the single-cell suspension through the magnetic column using MACS Columns (cat.# 130-041-202; Miltenyi Biotec, North Rhine-Westphalia, Germany). The acquired Lin^−^ cells were centrifuged at 2,000 r/min for 5 min, the supernatant was discarded, and the cells were resuspended. After that, 10 *μ*L Sca-1 (cat. # 108105; BioLegend) and 10 *μ*L c-Kit PE-c-Kit (cat. # 105807; BioLegend) antibodies were added and incubated on ice in the dark for 10 min. Last, the cell suspension was filtered through a 200-mesh sieve and subjected to cell sorting using a flow cytometer.

### 2.4. Coculture of ADSCs and LSK Cells

P3 ADSCs, and mouse fibroblasts (NIH3T3) (as control) were seeded at the density of 8 × 10^3^/cm^2^. At the confluence of 70%–80%, mitomycin C (50 ng/mL; cat.# M4287, Sigma–Aldrich, Darmstadt, Germany) was added and incubated for 12 hr. When the cell growth was stopped, the feeder cells were successfully prepared. The feeder cells were plated in 6-well plates (5 × 10^5^/well). After feeder cell adherence, 5 × 10^3^ LSK cells were cocultured with the feeder cells. The long-term culture medium (2 mL) (cat.# MyeloCult™ M5300, Stem cell Technologies, Vancouver, Canada) and hydrocortisone (10^−6^ M; cat.# 74142; Stem cell Technologies, Vancouver, Canada) were added to the coculture system [18]. The culture medium was replaced once a week. The coculture system was cultured in a 5% CO_2_ incubator at 37°C for 2 and 5 weeks. LSK cells were harvested and counted by flow cytometry.

To inhibit the activation of the Notch signal pathway, in another independent experiment, *γ*-secretase inhibitor (GSI) (10 mol/L) (S2188, Sigma–Aldrich, Darmstadt, Germany) [19] was added to the coculture system in the same environment as the experimental conditions described above.

Jagged-1 knockdown in ADSCs was achieved by siRNA [20]. When the confluence of ADSCs reached 80%, Jagged-1 siRNA (Biosyntech, Suzhou, China) was thoroughly mixed with lipofectamine 2000 (cat.#11668-027; Invitrogen, ThermoFisher, MA, USA). The final concentration of Jagged-1 siRNA was 10 nM. The transfection reagent was then cocultivated with ADSCs for 24 hr and subsequently discarded. PCR and protein detection of Jagged-1 were performed 48 hr after transfection. ADSCs with Jagged-1 knockdown were prepared as feeder layer cells by the same method and cultured with LSK cells under the same conditions for 2 and 5 weeks.

The cocultured LSK cells were isolated. LSK cells (1 × 10^3^) were seeded in 2 mL methylcellulose (cat.# 3434; Stem cell Technologies, Vancouver, Canada) and incubated at 37°C with 5% CO_2_ for 2 weeks [21]. The formation of cell colonies was observed under a microscope.

### 2.5. Detection with Scanning Electron Microscope

After coculturing for 5 weeks, the cocultured ADSCs and LSK cells were fixed with 2% glutaraldehyde for 12 hr. The samples were stored at 4°C overnight. Dehydration was performed sequentially with 50%, 70%, 80%, 90%, and 100% tert-butanol. The samples were frozen at −20°C, and then coated using a Sputter coater (SCD 050, BAL-TEC GmbH). The cell morphology was observed under a scanning electron microscope (PHILIPS XL 30 ESEM FEG; Philips, Eindhoven, The Netherlands) at an accelerating voltage of 10 kV.

### 2.6. Real-Time Quantitative PCR

The expressions of *Notch-1*, *Jagged-1*, and *Hes-1* mRNA in ADSCs, fibroblasts, LSK cells, and LSK cells after coculture were detected by real-time quantitative PCR. Total RNA was extracted from cells using Trizol (15596-018; Invitrogen, MA, USA). Reverse transcription PCR was performed with TaqMan® MicroRNA Reverse Transcription Kit (cat.#4366597; ThermoFisher, MA, USA). The RNA quantity for reverse transcription PCR was 2 ng, and the reaction system volume was 20 *μ*L. The reaction conditions were 42°C for 60 min; 75°C for 5 min, and 4°C for 5 min, one cycle. The primers for real-time quantitative PCR were designed and synthesized by Sangon Biotech (Shanghai, China). The primer sequences were *Jagged-1*, forward 5′- TCCAGGTCTTACCACCGAAC-3′ and reverse 5′- GACGCCTCTGAACTGAC-3′; *Notch-1*, forward 5′-GTGGTTCCCTGAGGGTTTCAA-3′ and reverse 5′-GGAACTTCTTGGTCTCCAGGT-3′; *Hes-1*, forward 5′-ACACGACACCGGACAAACCA-3′ and reverse 5′-TTATTCTTGCCCTTCGCCTCTT-3′; and, *β-actin*, forward 5′-ATCTACGAGGGCTATGCTCTCTCC-3′ and reverse 5′-CTTTGATGTCACGCACGATTTCC-3′. The PCR conditions were 95°C for 10 min; 95°C for 15 s, 55°C for 30 s, and 75°C for 30 s, for a total of 40 cycles. The cDNA volume was 2 *μ*L, and the reaction system volume was 20 *μ*L. The expression level of the target gene was determined by the ^*ΔΔ*Ct^ method.

### 2.7. Western Blot Analysis

Proteins were extracted from ADSCs, fibroblasts, LSK cells, and cocultured LSK cells after lysis with lysis buffer (cat.#78446; Thermo Scientific, Inc. MA, USA). Protein concentration was determined using a BCA Protein Assay kit (cat.# 23225; Thermo Scientific, Inc. MA, USA). The protein samples, each containing 20 *μ*g of protein, were subjected to 10% SDS-PAGE electrophoresis and the separated components were subsequently electrotransferred to a nitrocellulose membrane (cat. #24580; Cell Signaling Technology, Danvers, USA). Then, the membrane was blocked with 5% skim milk at room temperature for 2 hr, and probed with rabbit anti-Jagged-1 (1 : 1000; cat. # 70109, Cell Signaling Technology, Danvers, USA), rabbit anti-Notch-1 (1 : 2000; cat. # 36008, Cell Signaling Technology, Danvers, USA), and rabbit anti-Hes-1 (1 : 2000; cat. # 11988; Cell Signaling Technology, Danvers, USA) monoclonal antibodies. Then, goat anti-rabbit IgG (HRP) (1 : 1000; cat. # 3678; Cell Signaling Technology, Danvers, USA) was added for incubation at room temperature for 2 hr. The ECL kit (cat.#143237; Biosharp, Anhui, China) was used for detection. Grayscale values of target proteins were measured using Image Lab (version 6.1) software and normalized to those of *β*-actin.

### 2.8. Transplantation of ADSCs and LSK Cells in Mice

Experiments on animals were conducted in an SPF environment. After acclimation for 1 week, the NOD/SCID mice were subjected to irradiation with a linear accelerator [22] (irradiation dose nine Gry; 0.2 Gry/min). They were randomly divided into three groups utilizing the random number table method, with 10 mice in each group. The LSK group was infused with 1 × 10^3^ LSK cells. The LSK + fibroblasts group received an infusion of 1 × 10^3^ LSK cells and 1 × 10^5^ fibroblasts, and the LSK + ADSCs group was infused with 1 × 10^3^ LSK cells and 1 × 10^5^ ADSCs. The LSK cells, fibroblasts, and ADSCs were obtained from C57/BL mice (*n* = 10). Cells were administered via tail vein injection, with all cells being dispersed in a total volume of 200 *μ*L of sterile PBS. The blood routine test was performed weekly. The survival of mice was also observed.

### 2.9. Statistical Analysis

The data are expressed as mean ± SD. SPSS 17.0 software was used for statistical analysis. One-way ANOVA was used for multicomparison. Student-*t* test was used for intergroup comparison. Multiple linear regression analysis was employed to assess and compare the hematological recovery among the three distinct groups in the *in vivo* experiment. Survival was calculated using the Kaplan–Meier method, and the difference in survival was analyzed with the Log-Rank test. *P* < 0.05 was considered statistically significant. The graphs were plotted with Primes 6.0.

## 3. Results

### 3.1. Identification of ADSCs

**Figure 1:**
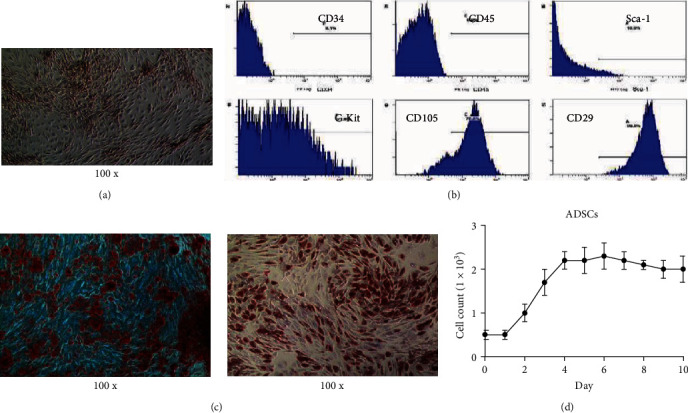
Isolation and identification of ADSCs. (a) The morphology of ADSCs. (b) Flow cytometry analysis of the surface markers of ADSCs, including CD105, CD29, CD34, and CD45. (c) The results of oil red O and alizarin red staining. (d) Growth curve of ADSCs.

The long-spindle typed ADSCs were observed, and there were swirl changes at the confluence of 80% (Figure 1(a)). Flow cytometry showed that there was CD29 and CD105 expression on ADSCs (Figure 1(b)). However, there was no expression of CD34 and CD45. This is consistent with the recognized immune phenotype of MSCs. Oil red O and alizarin red staining verified the multidirectional differentiation ability of ADSCs (Figure 1(c)). Cell growth curve showed that the ADSCs entered into an obvious logarithmic growth phase at 72 hr and entered a plateau phase after about 5 days (Figure 1(d)).

### 3.2. ADSCs Promote the Proliferation of LSK Cells In Vitro

**Figure 2:**
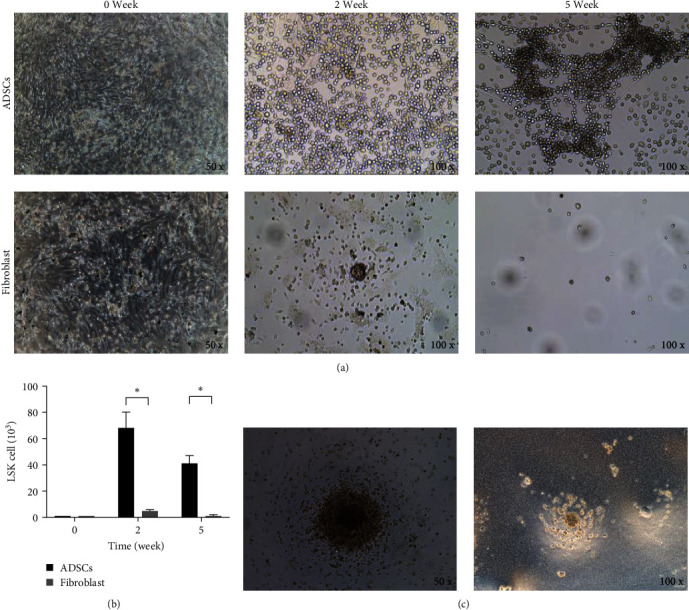
Effects of ADSCs on the proliferation of LSK cells *in vitro*. LSK cells were cocultured with ADSCs or fibroblasts. (a) In the coculture system, ADSCs could promote hematopoiesis of LSK cells. (b) The number of LSK cells at Day 0 and after coculture for 2 weeks and 5 weeks;  ^*∗*^*P* < 0.05. (c) Cell morphology after treatment with methylcellulose.

The LSK cells were cocultured with feeder cells prepared with ADSCs or fibroblasts. As shown in Figure 2(a), at 2 and 5 weeks, LSK cells cocultured with ADSCs still could proliferate and generate new cell colonies. However, this was not observed in LSK cells cocultured with fibroblasts. As shown in Figure 2(b), on Week 2 and Week 5, the number of LSK cells cocultured with ADSCs was significantly more than that cocultured with fibroblasts (ADSCs group: 6.8 ± 1.2 × 10^4^ at 2 weeks and 4.1 ± 0.6 × 10^4^ at 5 weeks; fibroblasts group: 5.0 ± 1 × 10^3^ at 2 weeks and 1 ± 0.2 × 10^3^ at 5 weeks; *P* < 0.05), suggesting that ADSCs can promote the proliferation of LSK cells *in vitro*.

The LSK cells obtained from the ADSCs coculture system were incubated with methylcellulose. After 2 weeks of culture, CFU-GM was observed under a microscope (Figure 2(c)). This result showed that LSK cells cocultured with ADSCs still maintained the ability of differentiation and proliferation even after long-term coculture.

### 3.3. The Relationship between ADSCs Feeder and LSK Cells Revealed by Scanning Electron Microscopy

**Figure 3:**
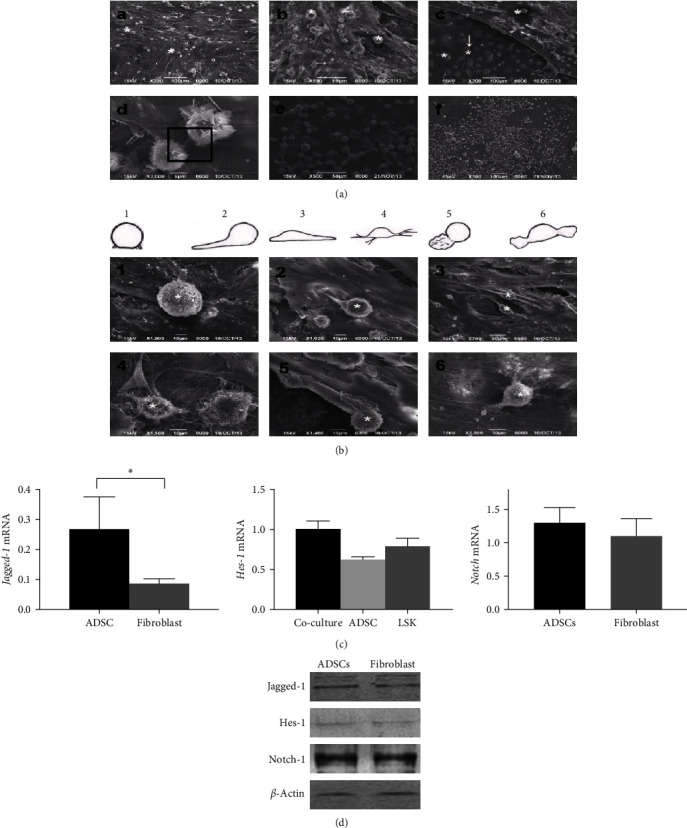
Notch signal pathway is activated when ADSCs are cocultured with LSK. ((a, b)) Scanning electron microscopy showing the direct cell–cell contact between ADSCs and LSKs at Week 2 (a) and Week 5 (b). (c) Real-time quantitative PCR analysis (upper panel) of *Jagged-1*, *Hes-1*, and *Notch*;  ^*∗*^*P* < 0.05. (d) Western blot analysis (lower panel) of Jagged-1, Hes-1, and Notch.

The relationship between LSK cells and the feeder layer was observed under a scanning electron microscope (Figures 3(a) and 3(b)). After coculture for 2 weeks, the morphology of LSK cells was mainly oval or spherical, which changed with the prolongation of culture time (Figure 3(a)). The pseudopodia of LSK cells increased, and filamentous and filamentous, and lamellar pseudopodia were observed. At Week 5 of coculture, LSK cells migrated to the bottom of the ADSCs feeder layer. These LSK cells and feeder cells were connected through pseudopodia and linear structures (Figure 3(b)).

### 3.4. The Notch Signal Pathway Is Activated during LSK Cell Proliferation

**Figure 4:**
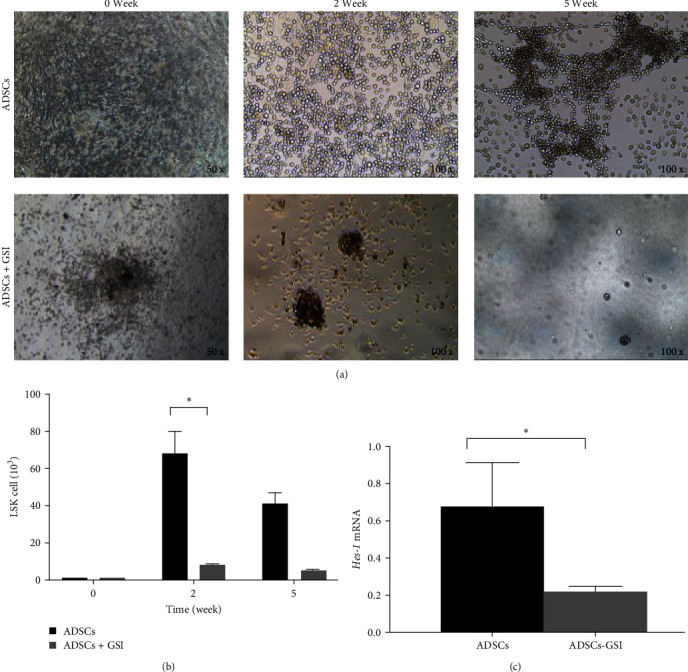
Effects of ADSCs on the proliferation of LSK cells *in vitro* with addition of GSI. (a) The cell morphology of coculture system. (b) The number of LSK cells at Day 0 and after coculture for 2 weeks and 5 weeks;  ^*∗*^*P* < 0.05. (c) Real-time quantitative PCR analysis of Hes-1 in LSK cells;  ^*∗*^*P* < 0.05.

Real-time quantitative PCR results showed that *Jagged-1* mRNA was expressed in both ADSCs and fibroblasts, and its level was significantly higher in ADSCs (Figure 3(c)). There were also *Notch-1* and *Hes-1* expressions in LSK cells. Western blot results showed the same trend (Figure 3(d)). At Week 5 of coculture, the mRNA of Hes-1 showed significant increases (*P* < 0.05), indicating the activation of the Notch signal pathway. Moreover, after treating coculture system with GSI, the number of LSK cells was significantly reduced at Week 2 (ADSCs group: 6.8 ± 1.2 × 10^4^ at 2 weeks and 4.1 ± 0.6 × 10^4^ at 5 weeks; ADSCs + GSI group: 8.2 ± 0.4 × 10^3^ at 2 weeks and 5.1 ± 0.6 × 10^3^ at 5 weeks; *P* < 0.05) (Figures 4(a) and 4(b)). The *Hes-1* mRNA level was also significantly decreased in LSK cells cocultured with ADSCs + GSI (Figure 4(c)). The above results reveal that the Notch signaling pathway plays an important role in the proliferation of LSK cells and that GSI can block the activation of Notch pathway and affect the proliferation of LSK cells.

### 3.5. ADSCs Promote LSK Cell Proliferation through Jagged-1

**Figure 5:**
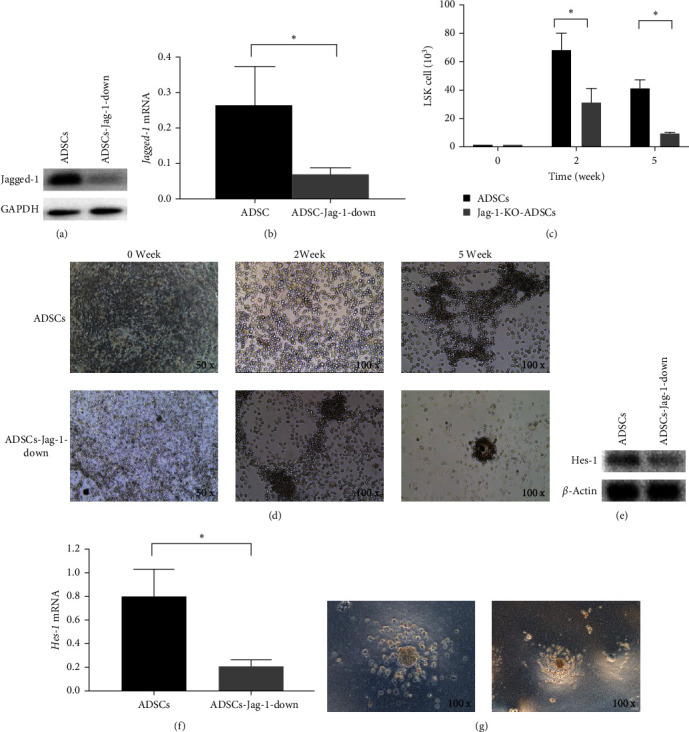
Effects of ADSCs on the proliferation of LSK cells *in vitro* after Jagged-1 knockdown. ADSCs were first subjected to Jagged-1 knockdown and then co-cultured with LSK cells. (a): Western blot analysis of Jagged-1 in ADSCs after Jagged-1 knockdown. (b): Real-time quantitative analysis of *Jagged*-1 in ADSCs after Jagged-1 knockdown. (c): The number of LSK cells at day 0 and after co-culture for 2 weeks and 5 weeks. (d): The cell morphology of coculture system. (e): Western blot analysis of Hes-1 in LSK cells. (f): Real-time quantitative analysis of *Hes-1* in LSK cells. (g): Hematopoietic colony morphology after cultured with methylcellulose.  ^*∗*^*P* < 0.05.

To explore the mechanism underlying the effects of ADSCs on LSK cell proliferation, Jagged-1 was knocked down in ADSCs by siRNA, and then coculture was conducted. Western blot (Figure 5(a)) and real-time quantitative PCR (Figure 5(b)) verified that Jagged-1 was efficiently knocked down in ADSCs. At Week 2 and Week 5 of coculture, the proliferation of LSK cells decreased significantly after the Jagged-1 knockdown (ADSCs group: 6.8 ± 1.2 × 10^4^ at 2 weeks and 4.1 ± 0.6 × 10^4^ at 5 weeks; Jagged-1-knockdown ADSCs group: 3.1 ± 1 × 10^4^ at 2 weeks and 9.2 ± 0.6 × 10^3^ at 5 weeks; *P* < 0.05) (Figures 5(c) and 5(d)). Similarly, the level of *Hes-1* of LSK also decreased after cocultured with the Jagged-1 knockdown ADSCs, indicating that the activation of the Notch signal pathway was weakened (Figures 5(e) and 5(f)). Additionally, after culture LSK with methylcellulose, CFU-GM was formed, but the ability to proliferate and differentiate was diminished (Figure 5(g)). These results show that Jagged-1 is the key factor for ADSCs to support LSK cells, but it may not be the only factor.

### 3.6. ADSCs May Promote Hematopoietic Recovery under the Condition of Insufficient LSK Cells

**Figure 6:**
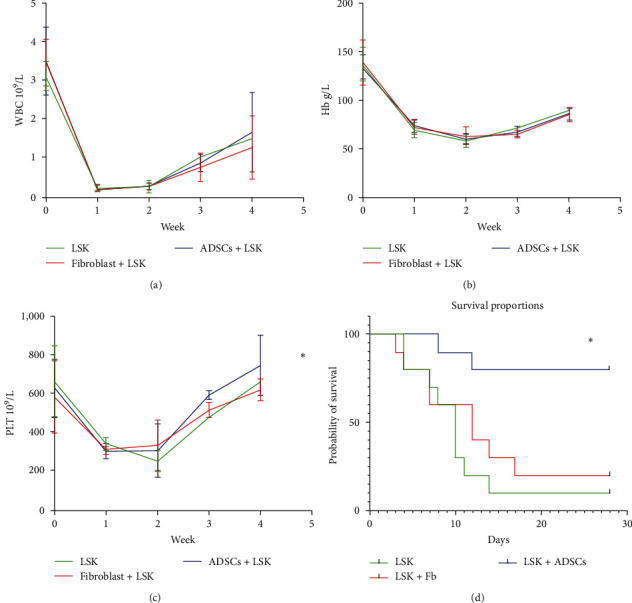
ADSCs support hematopoiesis *in vivo*. NOD mice received the infusion of LSK, LSK + fibroblasts, and LSK + ADSCs, respectively. (a) The alteration of white blood cells after transplantation. (b) The alteration of hemoglobin after transplantation. (c) The alteration of platelets after transplantation. (d) The survival rate of each group;  ^*∗*^*P* < 0.05.

After linear accelerated irradiation, NOD/SCID mice received the infusion of LSK, LSK + fibroblasts, and LSK + ADSCs, respectively. The number of LSK cells infused was lower than the number required for the recovery of hematopoietic function. The blood routine results showed that the leukocyte (Figure 6(a)), hemoglobin (Figure 6(b)), and platelets (Figure 6(c)) in the LSK + ADSCs group were higher than those in the other two groups at different times, indicating that ADSCs can support hematopoietic function, especially in platelets, although the results were not statistically significant in leukocytes and hemoglobin (Table 1). In terms of survival rate, eight mice in the LSK + ADSCs group survived more than 28 days. There were two survival mice in the LSK + fibroblasts group, and only one survival mouse in the LSK group. The median survival time was 28 days, 12 days, and 10 days, respectively (Figure 6(d), *P* < 0.01).

## 4. Discussion

With the booming of haploidentical HSCT, the incidence of PGF has been increasing, posing a challenge in clinical practice. A retrospective study found that 27.5% of patients were diagnosed with PGF [23]. Additionally, PGF patients had a higher risk of cytomegalovirus infection, further affecting their overall survival. Currently, there is no standardized treatment for PGF, and common clinical approaches include selected CD34^+^ cell infusion and the use of TPO-RA. Although CD34^+^ cell infusion has a high clinical response, it can also lead to serious graft-versus-host disease. In contrast, research on MSCs for treating PGF is ongoing. MSCs are important components of the bone marrow niche, play an important role in maintaining the proliferation and homeostasis of hematopoietic stem cells, and can improve the poor implantation after allogeneic HSCT [24, 25]. Previous studies have mostly focused on BMSCs [26, 27]. Muguruma et al. [28] reported that the injection of human BMSCs into the medullary cavity of NOD/SCID mice could promote the homing, proliferation, and differentiation of CD34^+^HSPCs (hematopoietic stem/progenitor cells) derived from human umbilical cord blood. In a study involving 20 patients [29], patients were treated with BMSCs at a dose of 1–3 × 10^6^/kg, of which 13 patients showed a positive treatment response, but there was also a higher rate of Epstein–Barr virus and cytomegalovirus infections.

Compared to current therapies for PGF, ADSCs offer several advantages. First, ADSCs have a stronger effect on supporting hematopoiesis both *in vitro* and *in vivo* compared to BMSCs. Particularly, ADSCs exhibit a superior long-term capacity for hematopoietic support [10]. Additionally, ADSCs have a lesser impact on the function of NK cells in comparison to BMSCs and umbilical cord-derived MSCs [8, 30, 31]. This suggests that the antiviral effect of NK cells could be better preserved during ADSC treatment for PGF. Furthermore, there may be residual tumor cells in PGF patients after HSCT, and excessive immune suppression may diminish the graft-versus-tumor (GVT) effect. Notably, NK cells are the main effector cells of the GVT effect. Since ADSCs have a weaker effect on NK cells, the GVT effect can be better preserved. Therefore, we suppose that ADSCs may be a promising alternative for PGF treatment.

During our investigation into the mechanisms of ADSCs in supporting hematopoiesis, we conducted a literature search on PUBMED from 2001 to the present. Although we found limited reports on this subject, several mechanisms were identified. In 2010, Nakao et al. [32] first reported the hematopoietic supportive role of ADSCs and suggested that they may enhance the homing of LSK cells by releasing CXCL-12. Another study by Ueda et al. [33] indicated that ADSCs had the potential to differentiate into osteoblasts and chondrocytes within the bone marrow niches, thereby supporting hematopoiesis. Furthermore, Foroutan et al. [34] found that ADSCs could regulate the proliferation and self-renewal of hematopoietic stem cells through miR-145. In this study, we detected the relationship between ADSCs and LSK cells using scanning electron microscopy. The results showed that the morphology of LSK cells varied when interacting with feeder cells. LSK cells had several types of plasma membrane processes, including microvilli, filaments, megapods, and cauda pods. Meanwhile, we observed elevated expression of *Hes-1* in LSK cells cocultured with ADSCs, indicating the activation of the Notch signal pathway. Therefore, we identified a novel mechanism suggesting that ADSCs may impact LSK cell proliferation via direct cell-to-cell contact. We demonstrate that this effect may be mediated by the Jagged-1/Notch-1/Hes-1 signaling pathway.

The Notch/Jagged signal pathway, a highly conserved pathway [35], is activated by close contact between cells. The signal transduction in the Notch/Jagged signal pathway is through protein hydrolysis of the Notch, but not phosphorylation [13]. After protein hydrolysis, the Notch protein fragments (NICD or ICN) with transcriptional activity would be released, which can then bind with the transcription factor CSL-DNA binding protein to regulate the expression of downstream genes. The *Hes-1* gene is one of the downstream genes necessary for the activation and function of the Notch/Jagged signal pathway [36]. The Notch/Jagged signal pathway is involved in different physiological and disease processes [37, 38]. Importantly, Jagged-1 is closely involved in hematopoietic function [39–42]. Duryagina et al. [39] showed that the loss of Jagged-1 in the endothelial cell from Jag1 ECKO mice led to the significant reduction of hematopoietic function and premature depletion of the adult hematopoietic stem cell pool. Notch-1 is detected in mouse hematopoietic cells [40]. Jagged-1 [41] was also found in the mesenchyme of the primary mouse fetal liver, which is the earliest place for hematopoiesis. LSK cells could maintain proliferative capacity after coculturing with 3T3 cells overexpressing human Jagged-1 [42]. These findings indicate the potential role of Notch ligands in the expansion of hematopoietic stem progenitor cells *in vitro* [43, 44]. In this study, we found that compared with mouse fibroblasts, the expression of Jagged-1 in ADSCs cells was higher. Moreover, the mRNA and protein expressions of *H*es*-1* in LSK cells cocultured with ADSCs were higher, suggesting that Notch is activated. Our results confirmed that ADSCs could activate the Notch signal pathway and promote the proliferation of LSK cells.

To further verify the role of the Notch signaling pathway in the proliferation of LSK cells, ADSCs were first treated with GSI [45] to inhibit Notch or transfected with Jagged-1 siRNA to knockdown Jagged-1, and then cocultured with LSK cells. We found that after treatment with GSI, the Notch pathway was not activated, and the proliferation ability of LSK was weakened. After the Jagged-1 knockdown, the hematopoietic support function of ADSCs was also weakened. These data indicate that the Jagged-1/Notch pathway importantly participates in the promoting effects of ADSCs on the proliferation of LSK cells. However, when Jagged-1 was deficient, some hematopoietic support function of ADSCs was maintained. The possible reason may be that the hematopoiesis is complex. The signal pathways such as Wnt-*β*- Catenin [46] and Hippo [47] play a regulatory role in hematopoiesis. Meanwhile, ADSCs can secrete a variety of important hematopoietic cytokines [48], such as LIF, M-CSF, G-CSF, and GM-CSF, which are importantly involved in hematopoiesis.

The role of ADSCs in supporting hematopoiesis *in vivo* has also been reported. As early as 2010, investigators [32, 49] infused ADSCs and LSK cells into NOD/SCID mice, and found that ADSCs could promote the homing and proliferation of LSK cells. In our previous study, we found that ADSCs could reduce acute graft-versus-host disease and promote further hematopoietic recovery in the mouse model with allogeneic transplantation [10]. In this study, a small dose of LSK cells was infused, which may cause poor implantation. Most mice in the LSK group and LSK + fibroblasts group failed to achieve hematopoietic recovery and eventually died. However, mice in the LSK + ADSCs achieved hematopoietic recovery, suggesting that ADSCs have strong supporting effects on hematopoiesis.

In future studies, there is great interest in enhancing the therapeutic potential of ADSCs in supporting hematopoiesis *in vivo*. Nanomaterials [50, 51] are expected to play a crucial role in addressing this challenge. On one hand, nanomaterials can act as carriers to guide the migration of ADSCs toward the bone marrow, thereby improving the microenvironment necessary for hematopoiesis. On the other hand, nanomaterials can modify the adhesion molecules on the surface of ADSCs, facilitating their interaction with hematopoietic stem cells [52]. This interaction enhances the signaling pathway mediated by Jagged-1/Notch-1/Hes-1, ultimately promoting hematopoietic function.

## 5. Conclusion

In our study, we confirm that ADSCs can support hematopoietic function *in vitro*. For the first time, we demonstrate that Jagged/Notch plays an important role in the promotive effect of ADSCs on LSK cell proliferation. Therefore, we propose that ADSCs may activate LSK cells through Jagged-1 and Notch-1, which may further activate the downstream transcription factors, and promote hematopoietic function. We believe that ADSCs may have the potential to improve PGF after HSCT.

## Figures and Tables

**Table 1:** Hematological recovery comparison among three experimental animal groups at different periods.

		0 Week	1 Week	2 Weeks	3 Weeks	4 Weeks	*F*	*P* value
Leukocyte (10^9^/L)	LSK	3.13 ± 0.63	0.26 ± 0.05	0.28 ± 0.15	1.05	1.52	2.509	0.120
	Fibroblast + LSK	3.48 ± 0.59	0.26 ± 0.08	0.28 ± 0.10	0.79 ± 0.37	1.30 ± 0.81		
	ADSCs + LSK	3.52 ± 0.86	0.22 ± 0.05	0.30 ± 0.11	0.88 ± 0.21	1.69 ± 1.03		

Hemoglobin (g/L)	LSK	136.00 ± 16.80	69.71 ± 7.71	58.20 ± 6.42	72.00	89.00	1.649	0.206
	Fibroblast + LSK	138.80 ± 22.84	70.80 ± 5.93	63.00 ± 9.30	65.00 ± 4.24	85.00 ± 7.07		
	ADSCs + LSK	134.00 ± 12.63	73.20 ± 7.12	59.60 ± 5.53	67.40 ± 5.32	85.4 ± 6.31		

Platelet (10^9^/L)	LSK	662 ± 183	341 ± 33	253 ± 57	478	660	5.419	0.025
	Fibroblast + LSK	584 ± 184	311 ± 22	333 ± 130	516 ± 38	622 ± 55		
	ADSCs + LSK	626 ± 145	299 ± 34	307 ± 137	595 ± 23	756 ± 155		

## Data Availability

The authors confirm that all data underlying the findings are fully available.
